# Book Reviews

**Published:** 1873-01-15

**Authors:** 


### Book Reviews.

The Pathology, Diagnosis and Treatment of Diseases of Women, including the Diagnosis of Pregnancy. By Graily Hewitt, M. D., L. and F. R. C. P.; Second American from the Third London Edition, Revised and Enlarged. Philadelphia: Lindsay & Blakiston.

This treatise is already well and favorably known to the American profession. It forms a volume of 740 pages, numerously illustrated. As regards this present edition, the author states in the preface that "It contains certain generalizations on the important questions of the pathology of diseases of the uterus, which have forced themselves on his attention in the course of several years' experience, and which involve the adoption of views in reference to the pathology and treatment of the diseases of the uterus which are new as compared with those embodied in the early editions of this work."

The mechanical system of uterine pathology now put forward is not, the author claims, a mere speculative theory. "If I had published it," he says, "when I first conceived it some years ago, it would have been a speculation only; but the system as now enunciated commends itself to my judgment as true, inasmuch as I have found it in conformity with daily observations for five or six years past. In support of these doctrines I have thought it expedient to embody the series of observations made by myself on the subject of the Diseases of Women at University College Hospital, during a period of over four years. These observations impart a clinical character to the work, which may be useful from other points of view."

These changes and additions which have been made, as well as the general rearrangement of the whole subject matter, render this new edition an essentially new work.

A Treatise on Diseases of the Nervous System. By William A. Hammond, M. D. Third Edition, with Additions and Corrections. New York: D. Appleton & Co.

The fact that this third edition of Prof. Hammond's valuable work has been called for within a year of its first issue, is the best possible evidence of the universal favor and appreciation with which it has been received by the profession. In the present edition the text has been thoroughly revised and a few additions made. The volume now comprises 750 octavo pages, illustrated by forty-eight well-executed engravings. As a thorough and complete treatise on the diseases of the nervous system, it occupies a field of its own, and forms an invaluable, indispensable addition to the physician's library.

